# Effects of stellate ganglion block on early brain injury in patients with subarachnoid hemorrhage: a randomised control trial

**DOI:** 10.1186/s12871-020-01215-3

**Published:** 2021-01-20

**Authors:** Jian Zhang, Ying Nie, Qiongni Pang, Xubiao Zhang, Qianting Wang, Jing Tang

**Affiliations:** 1 Department of Neurosurgery, 999 Brain Hospital, Guangzhou, 510515 Guangdong China; 2 Department of Anesthesiology, 999 Brain Hospital, Guangzhou, 510515 Guangdong China; 3 Department of Anesthesiology, Nanfang Hospital, Southern Medical University, Guangzhou, 510515 Guangdong China; 4 Department of Anesthesia, Affiliated Hospital of Guangdong Medical University, Zhanjiang, 524001 Guangdong China

**Keywords:** Early brain injury, Cervical sympathetic trunk, Stellate ganglion block, Subarachnoid hemorrhage, Transcranial Doppler

## Abstract

**Background:**

Subarachnoid hemorrhage (SAH) is a common neurosurgical emergency, and early brain injury (EBI) plays an important role in acute brain injury of SAH. Our objective is to investigate the effect of stellate ganglion block (SGB) on the clinical prognosis of patients with SAH (registration number ChiCTR2000030910).

**Methods:**

A randomized controlled trial was conducted with 102 participants. Patients with SAH were assigned to the SGB or nSGB group. Patients in the SGB group received SGB four times (once every other day starting on the day of the surgery). In contrast, patients in the nSGB group only received standard care. Data were collected on the day before surgery (T0) and on the 1^st^ (T1), 3^rd^ (T2) and 7^th^ day (T3) after surgery. The primary outcomes included EBI markers (including IL-1β, IL-6, TNF-α, ET-1, NPY, NSE and S100β), the mean cerebral blood flow velocity of the middle cerebral artery (Vm-MCA) and the basilar artery (Vm-BA). All cases were followed up for 6 months after surgery.

**Results:**

The levels of the EBI markers in both groups were higher at T1–T3 than at T0 (*P*<0.05), and the Vm-MCA and Vm-BA were also increased at the same times. However, the levels of the EBI markers were lower in the SGB group than in the nSGB group (*P*<0.05), and the increases of Vm-MCA and Vm-BA were also lower (*P*<0.05). The prognosis score and neurological deficit were better in the SGB group than in the nSGB group (*P*<0.05).

**Conclusions:**

SGB can improve the prognosis of SAH patients by inhibiting the inflammatory response during EBI and by reducing endothelial dysfunction and relieving CVS.

**Trial registration:**

Clinical trial number: ChiCTR2000030910; Registry URL: Chinese Clinical Trial Registry; Principal investigator's name: Ying Nie; Date of Trial registration: March, 2020 (retrospectively registered).

**Supplementary Information:**

The online version contains supplementary material available at 10.1186/s12871-020-01215-3.

## Background

Subarachnoid hemorrhage (SAH) is a neurosurgical emergency with a high morbidity and high mortality rate. Cerebral vasospasm is a common complication of SAH that develops as a sequelae to the hemorrhage and is one of the major contributors to mortality [1, 2]. Clinical trials based on preventing vasospasms, however, have, to date, achieved only limited success. The incidence of vasospasm is reduced without any reduction in delayed ischemic injury or improvements in long-term outcomes [3]. This fact has shifted research interest to the early brain injury (EBI) evoked by SAH.

In recent years, several pathological mechanisms that are activated within minutes after the initial bleed and that lead to EBI have been identified [4]. Current studies have shown that EBI plays an important role in acute brain injury of SAH within the first 72 hours. Initially, the direct damage to brain tissue caused by SAH within a very short period of time causes an increase in intracranial pressure and a decrease in cerebral blood flow (CBF), which in turn, lead to severe ischemia injury to brain tissue [5, 6]. Several inflammatory mediators contribute to SAH-induced cerebral inflammation and have a damaging effect on cerebral tissues, leading to neurobehavioral dysfunction, brain edema, blood-brain barrier (BBB) disruption, and neuronal cell apoptosis [7]. Moreover, brain-derived cytokines may enter systemic circulation in the presence of a post-SAH BBB disruption to activate inflammatory cascades systemically and contribute to the development of post-SAH systemic inflammatory response syndrome and extracerebral organ system failure [8, 9]. Researchers have confirmed that EBI has more important effects than CVS on the survival rate of SAH patients [4, 5, 10, 11]. This knowledge is beginning to transform experimental research on EBI into clinical applications, and it provides a new approach to the clinical treatment of SAH [5, 11–13].

Blocking the cervical sympathetic trunk is a treatment that was primarily used against pain-related diseases in the past. In recent years, this treatment has been used clinically to treat some diseases and has achieved good results. These diseases include traumatic brain injury and cerebral hemorrhage [14–18]. Cerebral blood vessels, in particular, pial vessels, have a dense nonadrenergic sympathetic nerve supply that originates mainly in the cervical ganglia and accompanies the carotid artery to project into the ipsilateral hemisphere [19, 20]. The intracerebral vessels constrict in response to cervical sympathetic stimulation and dilate when these fibers are interrupted [19, 20]. The release and reuptake of neurotransmitters, such as bradykinin, which is released during injury, can be prevented by sympathectomy [20].

The stellate ganglion is a fusion of the sympathetic ganglia at C7 and T1 and is the location used to apply a cervical sympathetic block. Although in 17% of the population there is no fusion at all in a strictly anatomical view, a cervical sympathetic trunk block is still referred to as a stellate ganglion block (SGB) in many studies. These studies indicated that SGB can prevent and relieve CVS by inhibiting cervical sympathetic nerves from dilating brain blood vessels and increasing the CBF, thereby improving microcirculation in the brain and metabolism in SAH patients, thus having a certain brain protection effect [18, 21, 22]. A recent report has suggested that blocking the cervical sympathetic trunk may be beneficial in patients with a subarachnoid hemorrhage [23]. In addition, our previous research also reported that SGB might have potential use in the treatment or control of cerebral vascular accidents in both elderly patients and patients with SAH [14]. These findings support the use of a superior cervical sympathetic trunk block to relieve CVS in patients with SAH.

However, neuroinflammation after SAH plays an important role in the pathophysiologic events of EBI, as mentioned above, and proinflammatory cytokines, such as IL-1β, IL-6, and TNF-α, are mediators of those immunologic reactions and are associated with the neurologic outcome [24, 25]. Therefore, we intend to further study the effect of SGB on the changes of inflammatory cytokine levels and cerebral blood vessels during EBI and to discuss the effect of SGB on the clinical prognosis of patients with SAH.

## Materials and Methods

All the patients who participated in this study signed an informed consent form to receive SGB. This study was conducted with the approval of the Institutional Ethics Review Board of GuangDong 999 Brain Hospital. This study was registered in the Chinese Clinical Trial Registry with the registration number ChiCTR2000030910 in March 2020.

This study was funded by the Science and Technology Program of Guangzhou. This study was approved by Guangzhou Science and Technology Information Bureau as early as July 2017 and was planned to be completed within three years. Unfortunately, the project funds were not in place until the end of 2018. According to the requirements of the hospital where the project was performed, the ethical approval process of the hospital can only be started when the funds are fully in place. Therefore, the project team began to apply to the hospital for ethical approval at the beginning of 2019. At the same time, as the end of the project was near, the project team could only recruit patients while applying. However, due to the slow approval process of the unit, all the approval processes were not completed until March 2020, so the clinical trial was registered later than the time of the patients entering the groups.

### Participants

#### Inclusion criteria

Participants who met the diagnostic criteria for aSAH ≥18 years old were recruited for this study.

#### Exclusion standard

**Fig. 1:**
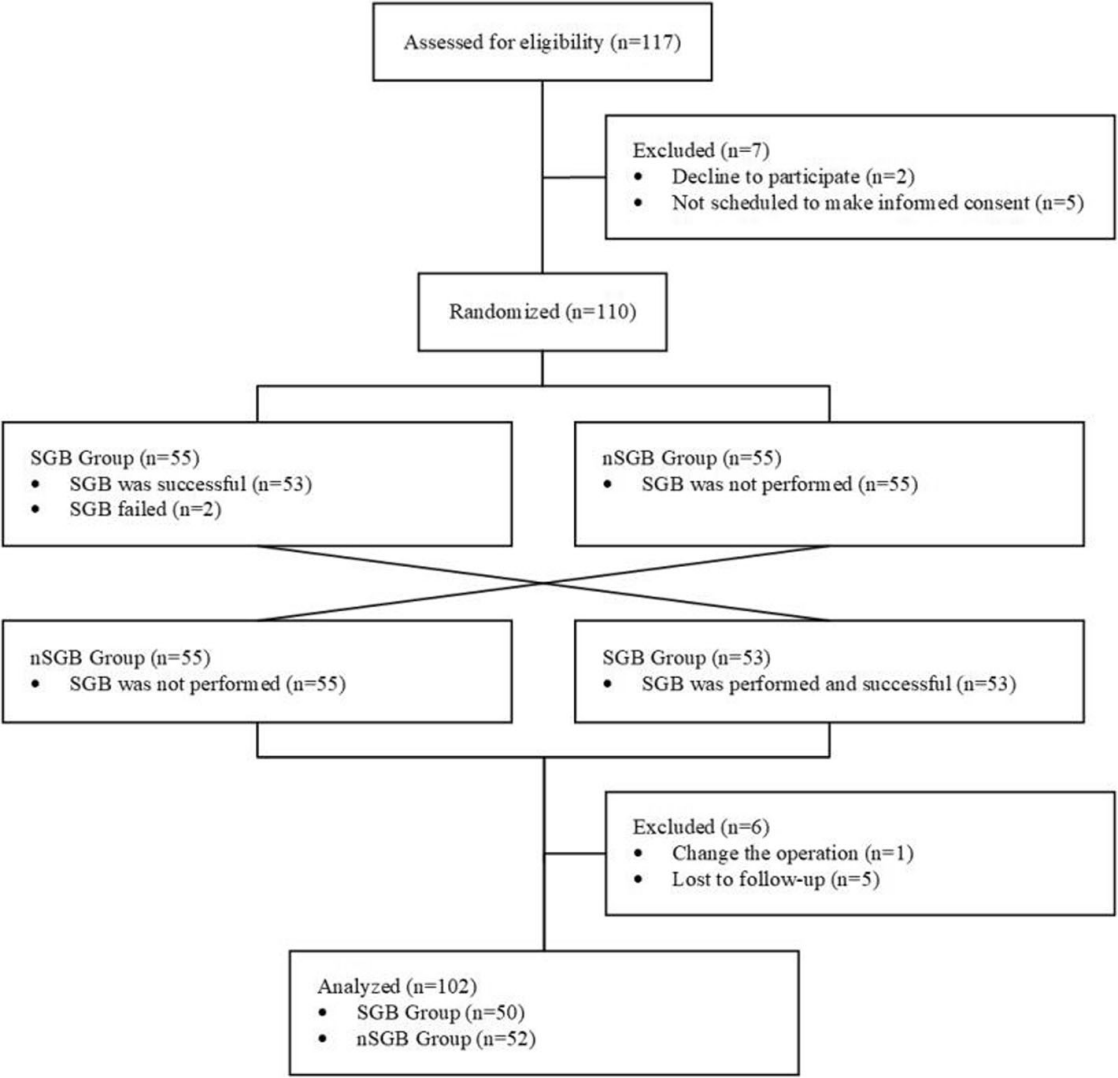
Flow chart showing the search process for included studies

Participants who met any of the following criteria were not eligible for this study:

- Participants<18 years old;
- Exclusion of an aneurysmal subarachnoid hemorrhage by CT scan;
- The time between the hemorrhage and surgery was more than 72 hours;
- A history of allergic reactions to local anesthetics;
- One or more other severe systemic diseases (including but not limited to tumors, cardiopulmonary insufficiency and hepatorenal insufficiency);
- Abnormal neck structure, such as scars and tumors that might interfere with the SGB treatment;

A coagulation disorder or undergoing anticoagulant therapy. The search process for included studies is shown in detai in Fig. 1.

### Materials and Methods 

#### Groups

Patients undergoing neurosurgical clipping were randomly divided into the SGB and nSGB groups (Supplemental Table 1*). Patients in the SGB group underwent SGB guided by ultrasound on the craniotomy side on the day of surgery (before anesthesia induction) and on the 2^nd^, 4^th^, and 6^th^ day after surgery (Supplemental Figure 5*), while patients in the nSGB group only received standard care. All patients were treated by the same operative anesthesia team.

A random number table was used to randomly distribute the patients into the groups. The person in charge of the research group generated the random allocation sequence, the secretary of the research group enrolled the participants, and the clinicians of the research group assigned the participants to the interventions. To achieve randomization, the work was relatively independent, meaning that those responsible for the assignment and registration were not allowed to participate in the trial intervention.

#### Sample size calculation

The necessary parameters needed to calculate the sample size were based on the results of our previous research published in the BJA article [14]. Our findings showed that the changes of Vm-MCA before and after the SGB block in the SAH group were 82.56 cm/s and 88.72 cm/s, respectively (P<0.005). Therefore, we inferred that SGB can induce a change of CBF in patients with SAH. According to these results, we assumed that the average Vm-MCA in the nSGB group was 82.56 cm/s, the average Vm-MCA in the SGB group was 88.72 cm/s, and the minimum necessary sample size of each group was 46 cases, 92 cases in total. The formula is as follows:

$$ {\displaystyle \begin{array}{l}{n}_c=\left(1+\raisebox{1ex}{$1$}\!\left/ \!\raisebox{-1ex}{$k$}\right.\right){\left(\mu 1-\alpha +\mu 1-\beta \right)}^2{\sigma}^2/{\left[\left( xT- xC\right)-\Delta \right]}^2\\ {}{n}_t={kn}_c\end{array}} $$

### Methods

#### The treatment protocols and quality control of aSAH patients

All patients were treated in accordance with the following to ensure their safety and a curative effect.

##### Preoperative preparation

All patients underwent a cranial computed tomography (CT) examination within 3 hours of arriving at the hospital and had the diagnosis of aSAH confirmed by computed tomographic angiography or digital subtraction angiography within 24 hours. Then, the grading scales, including Hunt-Hess, WFNS and Fisher, were completed according to the patient's examination results, and patients without any surgical contraindication were scheduled for surgery.

##### Anesthesia and surgery

All of the surgeries were performed in the operating room of our hospital with the same operative anesthesia team. Mean arterial blood pressure (MAP), central venous pressure and bispectral index (BIS) were digitally assessed before anesthesia induction, together with other physiological parameters, such as oxygen saturation and heart rate. Total intravenous anesthesia with propofol and sufentanil was administered to maintain the BIS values between 40 and 60. The fluctuations of MAP were maintained within 30% of the baseline values by vasoactive drugs. Monitoring of the motor evoked potential and somatosensory evoked potential was performed during the surgery. Craniotomy was performed through the pterion or the expanded pterion. During surgery, the aneurysms were located according to anatomic markers and exposed under a microscope, and then, a permanent aneurysm clamp was used to clamp the neck of the aneurysm. After clipping, the clipping effect was confirmed by angiography. The closure of the scalp was considered to be the end of surgery.

##### Patient management after surgery

All patients who underwent surgical clipping of aneurysms were treated in the postoperative period with a standard treatment protocol that included intensive care monitoring, maintaining normotension, fluid therapy to maintain normovolemia (positive fluid balance >500 ml/day), and spontaneous hemodilution to maintain a hematocrit of 30%. The axillary temperature (36.5°C–37.5°C) was maintained during the recovery period. Normal blood glucose (3.9 mmol/L–7.8 mmol/L) was usually controlled after surgery, and insulin could be used when needed. All patients received nimodipine 0.8–2.0 mg/h via intravenous micropumps. Then, HHH therapy was instituted. Hypervolemia was achieved with the administration of colloids and crystalloids with volume infusions of up to 3–4 L/day with a targeted central venous pressure of 10–12 mmHg. The MAP was targeted to 90–110 mmHg, which was achieved with the infusion of vasopressors (dopamine or noradrenaline). A CT scan of the brain was performed the first day after surgery and when the patient exhibited altered consciousness to determine whether there was cerebral edema, cerebral hemorrhage or cerebral vasospasm or other complications.

#### The methods of SGB

Patients were in the supine position with their head fixed in the middle, placing a thin pillow under the shoulder and neck if necessary. After aseptic skin preparation, a linear transducer (10 MHz) was placed on the neck to allow for cross-sectional visualization of the anatomical structure. First, the processus transversus of C7 was located, the probe direction and neck sagittal plane were placed at 45 degrees, and the probe was moved to confirm the location of the common carotid artery and stellate ganglion. The puncture needle was inserted from the lateral side of the probe to avoid the jugular vein and adjacent blood vessels and nerves, and then, it was moved to the stellate ganglion below the common carotid artery under the guidance of ultrasound. Ropivacaine (0.375%, 8 ml) was injected and the needle was adjusted to spread the liquid evenly. After the injection, the needle was pulled out. The signs of successful block were the appearance of Horner’s syndrome on the side of the injection, including contracted pupil, ptosis, enophthalmos, conjunctival hyperemia and facial reddishness without sweating. At the same time, possible complications of hematoma, pneumothorax, epidural or subarachnoid block, hoarseness, esophageal injury and thyroid injury were also observed and recorded.

#### Monitoring method of TCD

Vm-MCA and Vm-BA were monitored by a transcranial Doppler (TCD). The TCD monitor was from German DWL Company, Type BOX. A hand-held pulse probe (2 MHz) was used to explore the MCA from the temporal window and the BA from the occipital window. Monitoring was performed by one professional technician who did not know if the patient was undergoing SGB.

#### Measurement of markers of EBI

##### Collection and preservation of specimens

Patients were fasted for 2 hours, and then, 15 ml venous blood was collected from the internal jugular vein and placed in test tubes containing EDTA (the blood samples needed to be analyzed within 8 hours, or stored at 4 degrees for no more than 72 hours). The blood samples were centrifuged at 2000r/rain for 20 minutes to separate the serum. If the tests could not be immediately performed, then the serum was frozen at −20 degrees, and repeated freezing and thawing was avoided. All reagents were refrigerated at 2–8 degrees and allowed to equilibrate to room temperature for 30 minutes before use. Operating steps: the tests were performing using commercial kits following the manufacturer’s instructions. The results were recorded.

### Outcomes

#### Primary outcomes

Observation time point: the day before surgery (T0) and the 1^st^ day (T1), 3^rd^ day (T2) and 7^th^ day (T3) after surgery.

##### Monitoring of cerebral blood flow velocity

Observation index: Vm-MCA and Vm-BA (Supplemental Figure 5*).

##### EBI markers

Observation index: interleukin-1β (IL-1β), interleukin-6 (IL-6), tumor necrosis factor alpha (TNF-α), serum endothelin-1 (ET-1), neuropeptide (NPY), S100β protein (S100β), and neuron specific enolase (NSE) (Supplemental Figure 5*).

#### Secondary outcomes

Observation index: Glasgow Outcome score (GOS) and neurological injury (including hemiplegia, dysphasia and cognitive decline).

Observation time point: 6 months after surgery.

### Statistical analysis

Statistical analysis was performed using SPSS 22.0 (SPSS Institute, Chicago, IL, USA). Continuous variables are expressed as the mean with standard error (SD). Categorical variables are expressed as the frequency and percentage. Comparisons between groups were performed using the parametric t-test for continuous parameters and the Chi-square test or Fisher's exact test for categorical parameters. *P*<0.05 was considered statistically significant.

### Availability of data and materials

The datasets used and analysed during the study available from the corresponding author on reasonable request.

## Results

The 102 patients who underwent craniotomy for an intracranial aneurysm were randomly divided into the SGB and nSGB groups. There were no significant differences between the groups in their general characteristics (P_(age)_=.979, P_(sex)_=.239, P_(H-H)_=.727, P_(WFNS grade)_=.449, P_(Fisher grade)_=.554) (Table 1).

**Table 1:** Baseline Demographics and Clinical Features of Aneurysmal Subarachnoid Hemorrhage Patients

Variables	Overall (***N***=102)	SGB (***n***=50)	Non-SGB (***n***=52)	***P*** Value
**Age (years, mean±SD)**	51.96±2.71	51.43±2.22	53.76±1.74	0.979
**Sex (male, %)**	47 (46.1)	26 (52.0)	21 (40.4)	0.239
**Complication (%)**				0.547
hypertension	49 (48.0)	26 (52.0)	23 (44.2)	
diabetes	26 (25.5)	12 (24.0)	14 (26.9)	
other	7 (6.9)	3 (6.0)	4 (7.7)	
**H-H grade (%)**				0.727
I and II	69 (67.6)	33 (66.0)	36 (69.2)	
III and IV	33 (32.4)	17 (34.0)	16 (30.8)	
**WFNS grade (%)**				0.449
Good (I-II)	87 (85.3)	44 (88.0)	43 (82.7)	
Poor (III-IV)	15 (14.7)	6 (12.0)	9 (17.3)	
**Fisher grade (%)**				0.554
2	23 (22.5)	9 (18.0)	14 (26.9)	
3	43 (42.2)	22 (44.0)	21 (40.4)	
4	36 (35.3)	19 (38.0)	17 (32.7)	
**Aneurysm site (%)**				0.692
**ACoA**	11 (10.8)	7 (14.0)	4 (7.7)	
**ACA**	26 (25.5)	12 (24.0)	14 (26.9)	
**MCA**	47 (46.7)	22 (44.0)	25 (48.1)	
**PICA**	3 (2.9)	2 (2.0)	1 (1.9)	
**BA**	4 (3.9)	3 (6.0)	1 (1.9)	
**PCoA**	11 (10.8)	4 (4.0)	7 (13.5)	
**Time between admission and surgery (min, mean±SD)**	102	58.48±13.15	60.54±11.45	0.366
**Anesthesia time (min, mean±SD)**	102	343.15±32.68	369.49±41.14	0.402
**Operation time (min, mean±SD)**	102	218.57±29.78	231.71±20.54	0.824

Other complication includes hepatitis B, urinary stones. *H-H* Hunt-Hess, *WFNS* World Federation of Neurological Surgeons, *ACoA* anterior communicating artery; anterior cerebral artery, *MCA* middle cerebral artery, *PICA* posterior inferior cerebellar artery, *BA* basilar artery, *PCoA* posterior communicating artery

There was no significant difference in the general data of age and sex between SGB and Non-SGB group (*P*>0.05)

In the SGB group, there were no significant differences in the respiratory rate, SpO2 or MAP of patients after SGB. Transient hoarseness occurred in two patients but disappeared within 10 minutes. The other patients had no sensory or motor changes and no serious complications, such as a block of the phrenicus nerve.

### Comparison of the changes in CBF between the SGB and nSGB groups

**Fig. 2:**
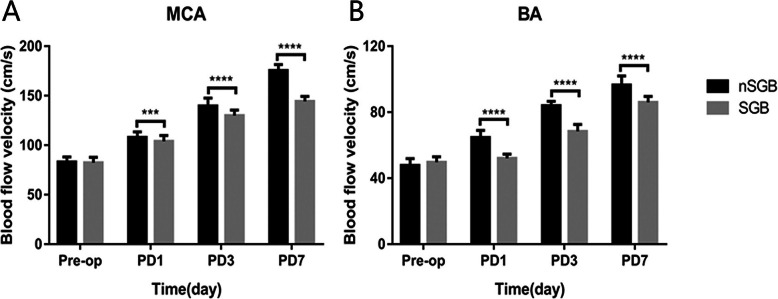
Changes in the Blood Flow Velocity of MCA and BA in Different Time Periods. Comparison of the changes in the blood flow velocity of MCA (**a**) and BA (**b**) in the different time periods. Each bar represents the mean±S.E.M. *** *P*<0.001, ****P<0.0001


There were no significant differences in Vm-MCA or Vm-BA between the SGB group and nSGB group at T0 (P_(Vm-MAC)_=.288, P_(Vm-BA)_=.309). Vm-MCA increased in both groups from T1–T3 compared to T0 (P_(PD1)_=.0006, P_(PD3)_ <.0001, P_(PD7)_ <.0001), and Vm-BA also increased in both groups from T1–T3 compared to T0 (P_(PD1)_ <.0001, P_(PD3)_ <.0001, P_(PD7)_ <.0001). However, the increases of Vm-MCA and Vm-BA from T1–T3 were lower in the SGB group than in the nSGB group (Fig. 2). In addition, Vm-MCA and Vm-BA in both groups were increased at T2 and reached a peak at T3. The peak value in the nSGB group was slightly more than a 100% increase compared to baseline. In the SGB group, there was a 50% increase, on average, and the lowest increase was approximately 20% (Supplemental Digital Content 2, 3 and 4).

**Additional file 4: Supplemental Digital Content 4.** The Classical case of mean blood flow velocities in each time and artery. **Supplemental Video 1.** The Classical case of Vm-MCA in SGB group-Before Operation.


**Additional file 5: Supplemental Digital Content 4.** The Classical case of mean blood flow velocities in each time and artery. **Supplemental Video 2.** The Classical case of Vm-MCA in SGB group-Post Operation Day1.


**Additional file 6: Supplemental Digital Content 4.** The Classical case of mean blood flow velocities in each time and artery. **Supplemental Video 3.** The Classical case of Vm-MCA in SGB group-Post Operation Day3.


**Additional file 7: Supplemental Digital Content 4.** The Classical case of mean blood flow velocities in each time and artery. **Supplemental Video 4.** The Classical case of Vm-MCA in SGB group-Post Operation Day7.


**Additional file 8: Supplemental Digital Content 4.** The Classical case of mean blood flow velocities in each time and artery. **Supplemental Video 5.** The Classical case of Vm-BA in SGB group-Before Operation.


**Additional file 9: Supplemental Video 6.** The Classical case of Vm-BA in SGB group-Post Operation Day1. (MP4 2425 kb)


**Additional file 10: Supplemental Digital Content 4.** The Classical case of mean blood flow velocities in each time and artery. **Supplemental Video 7.** The Classical case of Vm-BA in SGB group-Post Operation Day3.


**Additional file 11: Supplemental Digital Content 4.** The Classical case of mean blood flow velocities in each time and artery. **Supplemental Video 8.** The Classical case of Vm-BA in SGB group-Post Operation Day7.


**Additional file 12: Supplemental Digital Content 4.** The Classical case of mean blood flow velocities in each time and artery. **Supplemental Video 9.** The Classical case of Vm-MCA in nSGB group-Before Operation.


**Additional file 13: Supplemental Video 10.** The Classical case of Vm-MCA in nSGB group-Post Operation Day1.


**Additional file 14: Supplemental Digital Content 4.** The Classical case of mean blood flow velocities in each time and artery. **Supplemental Video 11.** The Classical case of Vm-MCA in nSGB group-Post Operation Day3.


**Additional file 15: Supplemental Digital Content 4.** The Classical case of mean blood flow velocities in each time and artery. **Supplemental Video 12.** The Classical case of Vm-MCA in nSGB group-Post Operation Day7.


**Additional file 16: Supplemental Digital Content 4.** The Classical case of mean blood flow velocities in each time and artery. **Supplemental Video 13.** The Classical case of Vm-BA in nSGB group-Before Operation.


**Additional file 17: Supplemental Digital Content 4.** The Classical case of mean blood flow velocities in each time and artery. **Supplemental Video 14.** The Classical case of Vm-BA in nSGB group-Post Operation Day1.


**Additional file 18: Supplemental Digital Content 4.** The Classical case of mean blood flow velocities in each time and artery. **Supplemental Video 15.** The Classical case of Vm-BA in nSGB group-Post Operation Day3.


**Additional file 19: Supplemental Digital Content 4.** The Classical case of mean blood flow velocities in each time and artery. **Supplemental Video 16.** The Classical case of Vm-BA in nSGB group-Post Operation Day7.

### Comparison of the changes of inflammatory cytokines in EBI between the SGB and nSGB groups

**Fig. 3:**
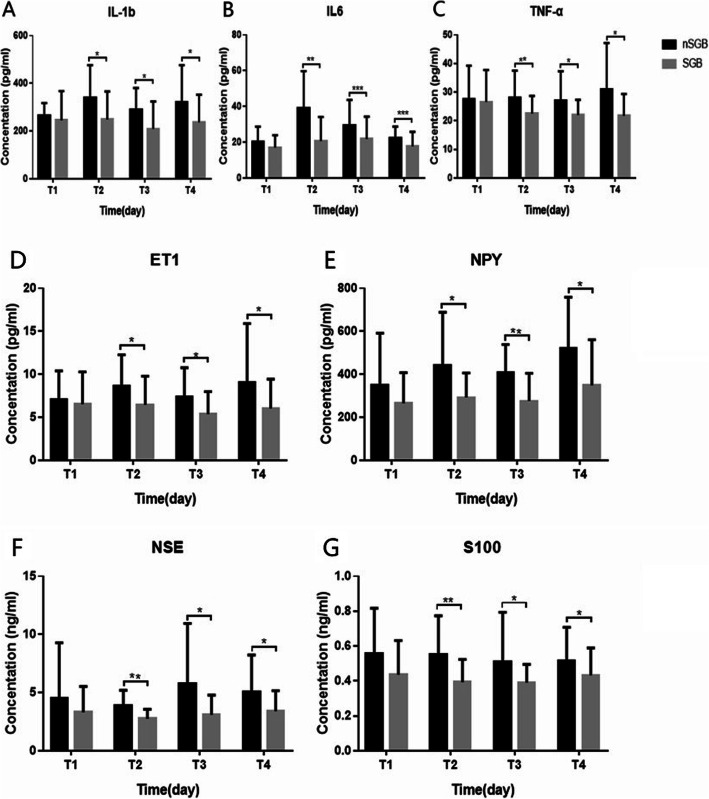
**a**-**c** Changes of Inflammatory Cytokines in EBI between the SGB Group and the nSGB Group. Comparison of the changes of inflammatory mediators such as Il-1β (**a**) Il-6 (**b**) and TNF-α (**c**) between the SGB Group and the nSGB Group. Each bar represents the mean±S.E.M. *P<0.05, **P<0.01*** *P*<0.001. **d**-**e** Changes of Vascular Physiological Markers in EBI between the SGB Group and the nSGB Group. Comparison of the changes of vascular physiological markers such as ET-1 (**d**) and NPY (**e**) between the SGB Group and the nSGB Group. Each bar represents the mean±S.E.M. *P<0.05, **P<0.01. **f**-**g** Changes of Brain Injury Markers in EBI between the SGB Group and the nSGB Group. Comparison of the changes of brain injury markers such as NSE (**f**) and S100β (**g**) between the SGB Group and the nSGB Group. Each bar represents the mean±S.E.M. *P<0.05, **P<0.01

There were no significant differences in the inflammatory cytokines levels between the SGB and nSGB groups at T0 (P_(IL-1β)_=.494, P_(IL-6)_=.143, P_(TNF-α)_=.782). The levels of cytokines increased in both groups from T1–T3 compared to T0. However, the increase of cytokines levels was lower in the SGB than in the nSGB group (PD1:P_(IL-1β)_=.0163, P_(IL-6)_=.0014, P_(TNF-α)_=.0448; PD3:P_(IL-1β)_=.0235, P_(IL-6)_=.0385, P_(TNF-α)_=.0430; PD7:P_(IL-1β)_=.0380, P_(IL-6)_=.0219, P_(TNF-α)_=.0224) (Fig. 3a-c).

### Comparison of the changes of vascular physiological markers in EBI between the SGB and the nSGB groups

There were no significant differences in the serum ET-1 or NPY levels between the SGB and nSGB groups at T0 (P_(ET-1)_=.626, P_(NPY)_=.169). The levels of vascular physiological markers increased in both groups from T1–T3 compared to T0. However, the increase of these markers was lower in the SGB group than in the nSGB group (PD1:P_(ET-1)_=.0356, P_(NPY)_=.0183; PD3:P_(ET-1)_=.0401, P_(NPY)_=.0061; PD7:P_(ET-1)_=.0477, P_(NPY)_=.0198) (Fig. 3d-e).

### Comparison of the changes of brain injury markers in EBI between the SGB and the nSGB groups

There were no significant differences in the serum NSE and S100β levels between the SGB and nSGB groups at T0 (P_(NSE)_=.277, P_(S100β)_=.067). The levels of brain injury markers increased in both groups from T1–T3 compared to T0. However, the increase of these markers was lower in the SGB group than in the nSGB group (PD1:P_(NSE)_=.0021, P_(S100β)_=.0032; PD3:P_(NSE)_=.0232, P_(S100β)_=.0420; PD7:P_(NSE)_=.0355, P_(S100β)_=.0225) (Fig. 3f-g).

### Comparison of the prognostic scores between the SGB and nSGB groups

The outcome parameters are presented in Table 2. The proportion of patients with a favorable clinical course outcome was 54% in the SGB group and 32.6% in the non-SGB group (P=.001). The proportion of patients with hemiplegia was 20% in the SGB group and 32.6% in the non-SGB group (P=.023).

**Table 2:** The difference in the recovery of the GOS scale between groups

Outcomes	Overall (***N***=102)	SGB (***n***=50)	Non-SGB (***n***=52)	***P*** value
**GOS (%)**				
I-II	18 (17.6)	7 (14.0)	11 (21.2)	0.063
III	40 (39.2)	16 (32.0)	24 (46.2)	0.027
IV-V	44 (43.1)	27 (54.0)	17 (32.6)	0.001
**Neurological deficit (%)**				
Hemiplegia	28 (27.5)	10 (20.0)	18 (34.6)	0.023
Cognitive decline	9 (8.8)	5 (10.0)	4 (7.7)	0.283
Dysphasia	5 (4.9)	2 (4.0)	3 (5.8)	0.539
Others	11 (10.8)	3 (6.0)	8 (15.4)	0.054

*GOS* Glasgow Outcome Scale; Others include epilepsy, hydrocephalus, and oculomotor nerve injury

## Discussion

EBI is thought to be an important cause of an unfavorable outcome after SAH [26]. Neuroinflammation and endothelial dysfunction are the two major mechanisms of EBI. Recent studies have demonstrated that neuroinflammation after SAH plays an important role in the pathophysiologic events of EBI. Many different inflammatory pathways are activated early in SAH, and early inflammation occurs mainly due to the proinflammatory cytokines IL-1β, IL-6 and TNF-α and other inflammatory chain-level reactions, resulting in a series of nervous system damage, such as a BBB disruption and brain edema, allowing blood-borne mononuclear cells and cytokines to enter the brain via paracellular routes [27, 28]. Brain-to-blood transport of some cytokines may also occur [29]. Increasing numbers of studies have found that an increased cytokine level is probably related to the intensity of SAH and secondarily aggravates vasospasm and ischemic changes in the brain [7, 9–11]. In addition, SAH can cause endothelial dysfunction immediately, and adverse factors, such as neuroinflammation and oxidative stress, promote the release of a large number of vasoconstrictors, such as ET-1, NPY [22, 30], and, especially IL-1β, which can induce the synthesis of additional ET-1, the strongest vasoconstrictor currently known. ET-1 can cause strong contractions of blood vessels, thus causing a loss of autonomic regulation of cerebral blood vessels, triggering cerebral vascular pathophysiology after SAH [31]. The roles played during the changing processes are important and are key factors that directly affect prognosis.

In this study, the levels of cytokines (IL-1β, IL-6 and TNF-α), vascular physiological (ET-1 and NPY) and brain injury markers (NSE and S100β) in both groups at each postoperative observation time point were all higher than those before surgery, and Vm-MCA and Vm-BA were also increased at the same time. TCD has been proven to be an ideal aid to monitor the effectiveness of various therapies instituted for vasospasm. The continuous increase of the cerebral blood flow velocity is closely related to vasospasm. When the cardiac output and blood pressure remain unchanged, the higher the increase, the more severe the spasm. The NSE and S100β proteins have been demonstrated to provide quantitative measures of brain damage and/or to improve the diagnosis and outcome evaluation in ischemic stroke, intracerebral hemorrhage, seizures, and comatose patients after cardiopulmonary resuscitation for cardiac arrest and traumatic brain injury [32–34]. Therefore, our findings are consistent with the conclusions of other researchers mentioned above, suggesting that an increase of cytokines levels is related to the development of CVS. This increase in inflammatory cytokines levels can be an indicator of injury to the central nervous system after an aSAH.

Recent studies have shown that targeted treatments for these mechanisms can effectively improve EBI and alleviate secondary damage after a cerebral hemorrhage [35]. Neuroinflammation is thought to be a promising area of research for new treatments [36–38]. SGB restrains the activity of the central and peripheral sympathetic nerves and corrects the pathological hyperfunction of sympathetic activity to restore normal levels and maintain homeostasis. Animal experiments indicate a tight connection between sympathetic nerves and inflammatory factors. Sympathetic nerve block reduces the concentrations of TNF-α, IL-1β, and IL-6 in SIRS [39]. In addition, SGB can prevent and relieve CVS by inhibiting cervical sympathetic nerves to dilate brain blood vessels and increase CBF. Our previous research also reported that SGB might have potential use in the treatment or control of cerebral vascular accidents in patients with SAH through its vasodilatory function [14].

Some previous studies have proven that the time course of cerebral vasospasm is unique in that it is slow developing, usually taking 4–7 days after SAH to peak [40]. In our study, Vm-MCA and Vm-BA in both groups were increased visibly on the 3rd day after surgery and reached their peaks on the 7th day after surgery. The peak value in the nSGB group was almost a 100% increase compared with baseline, higher than 100% in some patients. Therefore, our results are basically consistent with those of previous studies. However, in the SGB group, Vm-MCA and Vm-BA only showed a 50% increase on average, and the lowest increase was approximately 20%. At the same time, the levels of cytokines and vascular physiological markers at each postoperative observation time point were visibly lower in the SGB group than the nSGB group (*P*<0.05). NSE and S100β are the indexes used to determine the degree of damage in the early phase of trauma. The effect of SGB on the reduction of the NSE and S100β concentrations also appeared at each postoperative observation time point in the SGB group compared with the nSGB group (*P*<0.05), indicating that early treatment with SGB might further reduce nerve injury by inhibiting inflammation. To further clarify the influence of SGB on the prognosis of SAH patients, we followed up all of the subjects for 6 months and found that the prognosis score was better in the SGB group than the nSGB group (*P*<0.05) and that the incidence of postoperative dysfunction was lower (*P*<0.05), showing that early treatment with SGB might be important for the improvement of trauma prognosis.

The limitations of this study should be noted while interpreting our results. First, we did not measure the CSF concentrations of IL-1β, IL-6, TNF-α, ET-1, NPY, S100β and NSE at the same time. Therefore, we could not explain the detailed interactions of systemic inflammation between the peripheral and central nervous systems. Second, for economic reasons, we did not monitor intracranial pressure in every patient. A diagnostic brain computed tomographic (CT) scan was taken only when there was a clinical suspicion of vasospasm. This factor may influence our results. Third, the 8 mL volume may have led to the unavoidable spread of the local anesthetic to unwanted structures, such as the plexus cervicalis/brachialis. Although there were no significant complications in the SGB group, transient hoarseness occurred in two patients and disappeared within 10 minutes. Therefore, there may be some effects on vascular tone with such volumes.

## Conclusion

SGB can improve the prognosis of SAH patients by inhibiting the inflammatory response during EBI, thus reducing endothelial dysfunction and relieving CVS.

## Supplementary Information

Additional file 1: Supplemental Digital Content 1. Table 1*. Random Number of Patients allocation sequence.

Additional file 2: Supplemental Digital Content 2. Figure 1*. Classical Case of Mean Blood Flow Velocities of BA and MCA in SGB Group. The Vm-BA were monitored by TCD before surgery (A) and on the first day (B), the third day (C) and the seventh day (D) after surgery. The Vm-MCA in SGB group were monitored by TCD before surgery (E) and on the first day (F), the third day (G) and the seventh day (H) after surgery.

Additional file 3: Supplemental Digital Content 3. Figure 2*. Classical Case of Mean Blood Flow Velocities of BA and MCA in nSGB Group. The Vm-BA were monitored by TCD before surgery (A) and on the first day (B), the third day (C) and the seventh day (D) after surgery. The Vm-MCA in SGB group were monitored by TCD before surgery (E) and on the first day (F), the third day (G) and the seventh day (H) after surgery.

## Data Availability

The datasets used and/or analysed during the current study available from the corresponding author on reasonable request.

## Abbreviations

SAH: Subarachnoid hemorrhage
aSAH: Aneurysmal subarachnoid hemorrhage
EBI: Early brain injury
SGB: Stellate Ganglion Block
EBI: Early Brain Injury
MCA: Middle cerebral artery
BA: Basilar artery
Vm: Mean cerebral blood flow velocity
CVS: Cerebral vasospasm
CBF: Cerebral blood flow
BBB: Blood-brain barrier
CT: Computed tomography
TCD: Transcranial doppler
IL-1β: Interleukin-1β
IL-6: Interleukin-6
TNF-α: Tumor necrosis factor alpha
ET-1: Endothelin-1
NPY: Neuropeptide
S100β: S100βprotein
NSE: Neuron specific enolase
GOS: Glasgow Outcome score
H: Hunt-Hess
WFNS: World Federation of Neurological Surgeons
ACoA: Anterior communicating artery
ACA: Anterior cerebral artery
MCA: Middle cerebral artery
PICA: Posterior inferior cerebellar artery
BA: Basilar artery
PCoA: Posterior communicating artery
CN: Cranial nerve
