# Supplementary material for: Effects of stellate ganglion block on early brain injury in patients with subarachnoid hemorrhage: a randomised control trial
Source: BMC Anesthesiol. 2021 Jan 20;21:23. doi: 10.1186/s12871-020-01215-3 (PMC7816408; doi:10.1186/s12871-020-01215-3)
Supplement: Supplementary file 17 — Additional file 1: Supplemental Digital Content 1. Table 1*. Random Number of Patients allocation sequence. [file 12871_2020_1215_MOESM1_ESM.doc]

| fixed value：123456 | | |
| --- | --- | --- |
|  |  |  |
| patient | random number | group |
| 1 | 7390 | Non-SB |
| 2 | 2726 | SB |
| 3 | 7096 | Non-SB |
| 4 | 3192 | SB |
| 5 | 3679 | SB |
| 6 | 1046 | SB |
| 7 | 369 | SB |
| 8 | 5334 | Non-SB |
| 9 | 3714 | SB |
| 10 | 403 | SB |
| 11 | 7935 | Non-SB |
| 12 | 9591 | Non-SB |
| 13 | 6481 | Non-SB |
| 14 | 3392 | SB |
| 15 | 3374 | SB |
| 16 | 3781 | SB |
| 17 | 5611 | Non-SB |
| 18 | 9525 | Non-SB |
| 19 | 4829 | Non-SB |
| 20 | 7122 | Non-SB |
| 21 | 9316 | Non-SB |
| 22 | 1760 | SB |
| 23 | 8566 | Non-SB |
| 24 | 7250 | Non-SB |
| 25 | 445 | SB |
| 26 | 7123 | Non-SB |
| 27 | 8096 | Non-SB |
| 28 | 607 | SB |
| 29 | 1850 | SB |
| 30 | 5625 | Non-SB |
| 31 | 6048 | Non-SB |
| 32 | 2454 | SB |
| 33 | 9055 | Non-SB |
| 34 | 4449 | SB |
| 35 | 1068 | SB |
| 36 | 9437 | Non-SB |
| 37 | 5347 | Non-SB |
| 38 | 2164 | SB |
| 39 | 5308 | Non-SB |
| 40 | 5155 | Non-SB |
| 41 | 6466 | Non-SB |
| 42 | 1250 | SB |
| 43 | 3607 | SB |
| 44 | 1336 | SB |
| 45 | 5269 | Non-SB |
| 46 | 7318 | Non-SB |
| 47 | 7088 | Non-SB |
| 48 | 1845 | SB |
| 49 | 2606 | SB |
| 50 | 7026 | Non-SB |
| 51 | 3152 | SB |
| 52 | 6834 | Non-SB |
| 53 | 2929 | SB |
| 54 | 2927 | SB |
| 55 | 6607 | Non-SB |
| 56 | 327 | SB |
| 57 | 982 | SB |
| 58 | 414 | SB |
| 59 | 4555 | SB |
| 60 | 6583 | Non-SB |
| 61 | 2129 | SB |
| 62 | 5078 | Non-SB |
| 63 | 9723 | Non-SB |
| 64 | 2857 | SB |
| 65 | 2476 | SB |
| 66 | 5207 | Non-SB |
| 67 | 819 | SB |
| 68 | 6200 | Non-SB |
| 69 | 6398 | Non-SB |
| 70 | 407 | SB |
| 71 | 423 | SB |
| 72 | 7264 | Non-SB |
| 73 | 8759 | Non-SB |
| 74 | 3809 | SB |
| 75 | 2149 | SB |
| 76 | 9046 | Non-SB |
| 77 | 1095 | SB |
| 78 | 3339 | SB |
| 79 | 9664 | Non-SB |
| 80 | 5394 | Non-SB |
| 81 | 7819 | Non-SB |
| 82 | 4840 | Non-SB |
| 83 | 5394 | Non-SB |
| 84 | 1828 | SB |
| 85 | 8726 | Non-SB |
| 86 | 3312 | SB |
| 87 | 702 | SB |
| 88 | 2801 | SB |
| 89 | 1195 | SB |
| 90 | 6983 | Non-SB |
| 91 | 1781 | SB |
| 92 | 1588 | SB |
| 93 | 6985 | Non-SB |
| 94 | 2798 | SB |
| 95 | 482 | SB |
| 96 | 9316 | Non-SB |
| 97 | 9674 | Non-SB |
| 98 | 5754 | Non-SB |
| 99 | 6606 | Non-SB |
| 100 | 3191 | SB |

**Supplemental Table 1.Random Number of Patients** **allocation sequence.**
