# Supplementary figures and images for: Effects of stellate ganglion block on early brain injury in patients with subarachnoid hemorrhage: a randomised control trial
Source: BMC Anesthesiol. 2021 Jan 20;21:23. doi: 10.1186/s12871-020-01215-3 (PMC7816408; doi:10.1186/s12871-020-01215-3)

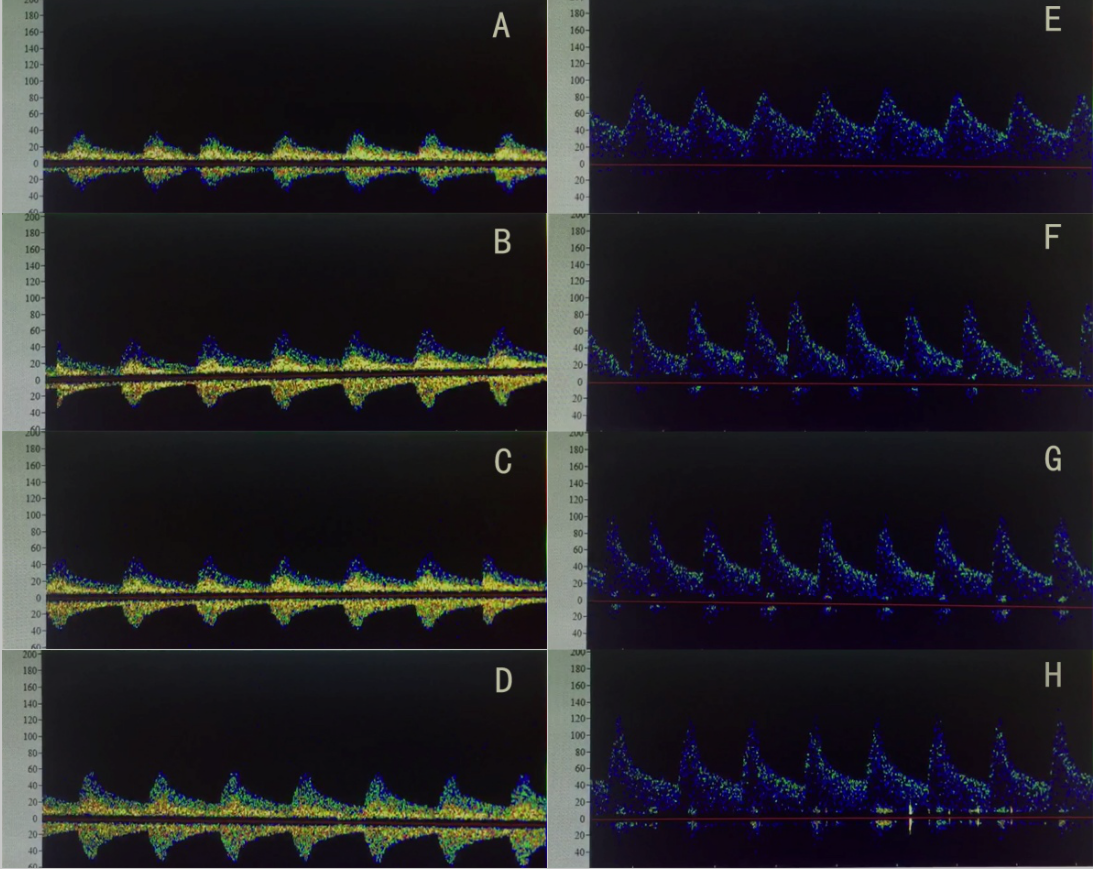

Supplement: Supplementary file 18 — Additional file 2: Supplemental Digital Content 2. Figure 1*. Classical Case of Mean Blood Flow Velocities of BA and MCA in SGB Group. The Vm-BA were monitored by TCD before surgery (A) and on the first day (B), the third day (C) and the seventh day (D) after surgery. The Vm-MCA in SGB group were monitored by TCD before surgery (E) and on the first day (F), the third day (G) and the seventh day (H) after surgery. [file 12871_2020_1215_MOESM2_ESM.tif]

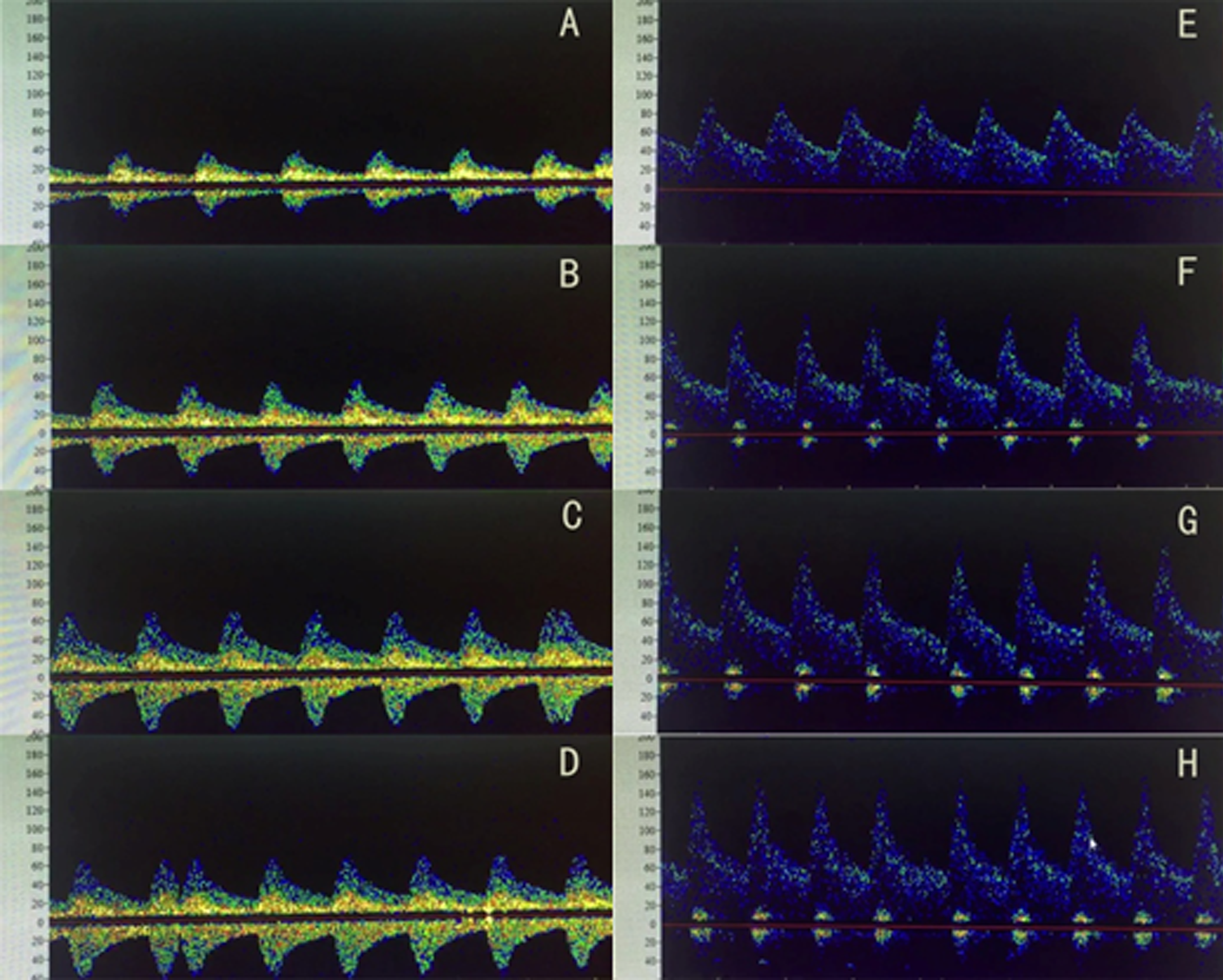

Supplement: Supplementary file 19 — Additional file 3: Supplemental Digital Content 3. Figure 2*. Classical Case of Mean Blood Flow Velocities of BA and MCA in nSGB Group. The Vm-BA were monitored by TCD before surgery (A) and on the first day (B), the third day (C) and the seventh day (D) after surgery. The Vm-MCA in SGB group were monitored by TCD before surgery (E) and on the first day (F), the third day (G) and the seventh day (H) after surgery. [file 12871_2020_1215_MOESM3_ESM.tif]
